# Describing the characteristics and healthcare use of high-cost acute care users at the end of life: a pan-Canadian population-based study

**DOI:** 10.1186/s12913-020-05837-8

**Published:** 2020-10-31

**Authors:** Danial Qureshi, Sarina Isenberg, Peter Tanuseputro, Rahim Moineddin, Kieran Quinn, Christopher Meaney, Kimberlyn McGrail, Hsien Seow, Colleen Webber, Robert Fowler, Amy Hsu

**Affiliations:** 1 Clinical Epidemiology Program, Ottawa Hospital Research Institute, Ottawa, ON Canada; 2 Bruyère Research Institute, Ottawa, ON Canada; 3 Institute of Health Policy, Management and Evaluation, University of Toronto, Toronto, ON Canada; 4 Temmy Latner Centre for Palliative Care and Lunenfeld Tanenbaum Research Institute, Sinai Health System, Toronto, ON Canada; 5 Department of Medicine, Division of Internal Medicine, University of Toronto, Toronto, ON Canada; 6 Centre for Health Services and Policy Research, Faculty of Medicine, The University of British Columbia, Vancouver, BC Canada; 7 Department of Oncology, Faculty of Health Sciences, McMaster University, Hamilton, ON Canada; 8 Department of Medicine, Interdepartmental Division of Critical Care Medicine, University of Toronto, Toronto, ON Canada

**Keywords:** Acute care, High-cost user, End of life

## Abstract

**Background:**

A minority of individuals use a large portion of health system resources, incurring considerable costs, especially in acute-care hospitals where a significant proportion of deaths occur. We sought to describe and contrast the characteristics, acute-care use and cost in the last year of life among high users and non-high users who died in hospitals across Canada.

**Methods:**

We conducted a population-based retrospective-cohort study of Canadian adults aged ≥18 who died in hospitals across Canada between fiscal years 2011/12–2014/15. High users were defined as patients within the top 10% of highest cumulative acute-care costs in each fiscal year. Patients were categorized as: persistent high users (high-cost in death year and year prior), non-persistent high users (high-cost in death year only) and non-high users (never high-cost). Discharge abstracts were used to measure characteristics and acute-care use, including number of hospitalizations, admissions to intensive-care-unit (ICU), and alternate-level-of-care (ALC).

**Results:**

We identified 191,310 decedents, among which 6% were persistent high users, 41% were non-persistent high users, and 46% were non-high users. A larger proportion of high users were male, younger, and had multimorbidity than non-high users. In the last year of life, persistent high users had multiple hospitalizations more often than other groups. Twenty-eight percent of persistent high users had ≥2 ICU admissions, compared to 8% of non-persistent high users and only 1% of non-high users. Eleven percent of persistent high users had ≥2 ALC admissions, compared to only 2% of non-persistent high users and < 1% of non-high users. High users received an in-hospital intervention more often than non-high users (36% vs. 19%). Despite representing only 47% of the cohort, persistent and non-persistent high users accounted for 83% of acute-care costs.

**Conclusions:**

High users – persistent and non-persistent – are medically complex and use a disproportionate amount of acute-care resources at the end of life. A greater understanding of the characteristics and circumstances that lead to persistently high use of inpatient services may help inform strategies to prevent hospitalizations and off-set current healthcare costs while improving patient outcomes.

## Background

A relatively small number of individuals use a large portion of health system resources. Estimates from Canada and the US show that the top 5 to 10% of healthcare users account for greater than 50% of healthcare use and associated costs [1–6]. A recent study by Wodchis et al. found that acute-care costs account for more than 30% of total healthcare expenditures for the top decile of users [7]. These “high users” often have significant healthcare needs [8–11].

As health systems and policymakers continue to strive towards achieving effective cost-saving strategies while improving patient outcomes, a better understanding of patients’ trajectories of high-cost acute-care use – particularly at the end of life – has become a pressing concern.

The end-of-life period is known to be associated with disproportionately high cost [12]. Past research has shown that among high-cost patient populations, substantial variation exists in terms of demographics, diagnoses, disease and overall healthcare use [13–17]. Despite the considerable impact this small population has on healthcare systems, little work has been done to describe their characteristics and inpatient healthcare use by their trajectory of acute-care use as they approach death. One Canadian study compared the characteristics and inpatient healthcare use among several high-cost inpatient groups consisting of survivors and decedents in an acute tertiary hospital, but none have examined these trends at the national level nor focused on the end-of-life period [18]. The majority of the work evaluating high users is limited to US Medicaid and Medicare populations and/or those aged ≥65 years old [19]. Research examining the demographic, clinical characteristics and patterns of healthcare use among high-cost end-of-life acute-care users is limited; a better understanding of these trends is vital for identifying opportunities for potential upstream prevention efforts to avoid acute-care use where possible, which may result in better health outcomes for these vulnerable populations.

Therefore, we conducted a population-based retrospective cohort study of Canadians who died in hospital. Specifically, we describe and contrast characteristics as well as inpatient healthcare use and acute-care expenditures in the last year of life across three cost groups: persistent high users, non-persistent high users and non-high users of acute-care.

## Methods

### Study population

We captured all Canadian adults aged 18 or older who died in an inpatient setting between fiscal years 2011/12 and 2014/15. The fiscal year period spans from April 1st of 1 year to March 31st of the following year. Individuals were excluded if they did not have at least two consecutive years of available data (i.e., the fiscal year of death, and year prior to death); had erroneous or missing data (i.e., invalid health card number or missing age and/or sex); were not a Canadian resident (identified by method of payment); had an acute-care length of stay ≥360 days (as these admissions are not representative of typical acute-care admissions); had an inpatient record for which a resource intensity weight (RIW) was not assigned (meaning there was no ability to calculate costs); or, had multiple death dates (more information available in Supplemental Table 1). We also chose to refrain from any discussion pertaining to findings from the Canadian territories (i.e., Yukon, Northwest Territories, and Nunavut – which are included in our analysis) due to their small sample sizes.

### Data sources

The primary data source for this study was the Discharge Abstract Database (DAD), which contains demographic, administrative, and clinical information on patients discharged from public hospitals in Canada (excluding Quebec, due to unavailable hospitalization data) [20]. Then, using encrypted identifiers – which were assigned based on patient health card numbers, province of residence and birth year – hospital records (from over 550 hospitals across Canada) from the DAD were linked to the Canadian Institute for Health Information’s (CIHI) Dynamic Cohort of Complex, High System Users. The Dynamic Cohort is an inpatient dataset that contains several subset cohorts encompassing various definitions of “complex, high system users” [21]. In this analysis, we used a subset cohort defined based on the highest acute-care costs; these high users were defined as the top 10% of highest cumulative acute-care cost patients in each fiscal year (more information in Supplemental Table 2). Briefly, inpatient hospitalization costs for each episode of care were derived by multiplying the province-specific Cost of Standard Hospital Stay value by the RIW for each record in the DAD.

### Case definition

We grouped patients into categories based on their pattern of acute-care cost across 2 years (fiscal year of death and year prior to death): persistent high users (high-cost in year prior to death and in death year), non-persistent high users (high-cost in death year only) and non-high user (never high-cost). Note that we do not discuss findings of those flagged as ‘high-cost in the year prior to death, but not in the death year’ (who represent 7% of the study population). As indicated by previous research [7, 18], it is likely that these patients had access to and received services provided in non-hospital sectors, which resulted in their reduced use of hospital-based services. Without this additional information, results from this group were difficult to interpret. Nonetheless, characteristics of patients in this category are presented in Supplemental Tables 3–5.

### Statistical analysis

Descriptive statistics were used to describe and contrast patients’ characteristics, their inpatient healthcare use and acute-care costs across our three comparison groups. Costs were inflated to 2014 Canadian dollars using Statistics Canada’s Consumer Price Index for health and personal care [22]. We described the study population according to their baseline characteristics in the fiscal year of death, including their age, sex, rurality, Elixhauser chronic conditions [23, 24], and number of comorbidities. Additionally, we examined their acute-care use in the last year of life, including the number of admissions and length of stay in hospitals, admissions to an intensive care unit (ICU), placement in an alternate-level-of-care (ALC) designated bed, terminal hospitalization admission type, and receipt of in-hospital interventions (mechanical ventilation, cardiopulmonary resuscitation, defibrillation, dialysis, percutaneous coronary intervention, feeding tube, blood transfusion, bronchoscopy). An ALC designation is provided to individuals occupying an acute care bed who have been medically cleared for hospital discharge, but remain in the hospital setting due to lack of availability of appropriate alternatives that would allow for a safe discharge (e.g., personal care, homecare, and long-term care services) [25]. Furthermore, a colour-coded map was used to display differences in the proportion of high users (persistent plus non-persistent) across Canada and differences in their median ICU and ALC lengths of stay in the last year of life (where data was available). All analyses were performed using SAS version 9.4 (SAS Institute, Cary, North Carolina).

## Results

### Cohort characteristics

We identified 191,310 Canadian adults who died in a hospital between fiscal years 2011/12 and 2014/15; among which 6% were persistent high users, 41% were non-persistent high users, and 46% were non-high users. High users (persistent plus non-persistent) differed substantially from non-high users in demographic and clinical characteristics (Table 1). Compared to non-high users, high users were younger (23% vs. 17% under 65 years old) and more likely to be male (55% vs. 51%). Overall, most patients resided in urban areas (88%); this did not seem to vary considerably across groups. In terms of their health profile, high users were more likely to have multimorbidity (48% vs. 35% had ≥3 co-occurring conditions) as well as higher rates of several specific chronic conditions, including congestive heart failure, chronic obstructive pulmonary disease, depression, renal failure and being a complex diabetic patient. Comparing among high users, persistent high users had a higher rate of complex diabetes (31 vs. 23%), congestive heart failure (27 vs. 23%), renal failure (14 vs. 10%), and chronic obstructive pulmonary disease (19% vs 16%) than non-persistent high users.

**Table 1:** Cohort characteristics by high user groups

	Persistent high user (***N*** = 11,375)		Non-persistent high user (***N*** = 78,989)		Non-high user (***N*** = 88,141)		Total^**a**^ (***N*** = 191,310)	
Characteristics	N	Col%	N	Col%	N	Col%	N	Col%
**Sex**								
Female	5025	44	35,846	45	42,829	49	89,751	47
Male	6350	56	43,143	55	45,312	51	101,559	53
**Age**								
18–44	628	6	2297	3	1885	2	5152	3
45–54	948	8	4621	6	3773	4	10,021	5
55–64	2019	18	10,592	13	8955	10	23,144	12
65–74	2756	24	17,392	22	16,008	18	38,787	20
75–84	3125	27	24,036	30	25,724	29	56,831	30
≥ 85	1899	17	20,051	25	31,796	36	57,375	30
**Rurality**								
Urban	10,052	88	69,443	88	76,938	87	167,666	88
Rural	1323	12	9546	12	11,203	13	23,644	12
**Selected Elixhauser Comorbid Conditions**								
Cardiac Arrhythmia	2348	21	17,991	23	15,642	18	38,232	20
Congestive Heart Failure	3036	27	18,510	23	16,400	19	40,917	21
Chronic Obstructive Pulmonary Disease	2213	19	12,642	16	13,212	15	30,424	16
Depression	410	4	2227	3	1202	1	4037	2
Diabetes - Complicated	3575	31	17,933	23	15,455	18	40,037	21
Diabetes - Uncomplicated	721	6	5062	6	5611	6	12,248	6
Hypertension - Complicated	133	1	744	1	430	< 1	1419	1
Hypertension - Uncomplicated	2412	21	19,606	25	22,240	25	47,180	25
Hypothyroidism	302	3	1815	2	1644	2	4001	2
Liver Disease	1033	9	6409	8	4560	5	12,661	7
Lymphoma	480	4	2884	4	1867	2	5657	3
Metastatic Cancer	1707	15	15,755	20	18,901	21	38,802	20
Other Neurological Disorders	963	8	6888	9	5785	7	14,431	8
Psychoses	84	1	463	1	316	< 1	913	< 1
Renal Failure	1596	14	7886	10	6959	8	17,785	9
Tumor (Solid Tumor without Metastasis)	2439	21	21,449	27	24,181	27	51,253	27
**# of Comorbidities**								
0	675	6	5071	6	9500	11	16,464	9
1–2	5152	45	36,516	46	47,903	54	96,242	50
3–5	5032	44	34,520	44	29,144	33	73,304	38
6+	516	5	2882	4	1594	2	5300	3

^a^total denominator includes those flagged as ‘high-cost in the year prior to death, but not in the death year’ (represent 7% of the study population)

### Inpatient healthcare use

In the last year of life, high users were more likely to have ≥3 hospital admissions (Table 2) than non-high users; specifically, persistent high users were more likely to experience multiple hospitalizations than non-persistent high users and non-high users (≥3 admissions: 85% vs. 48% vs. 21%, respectively). The median number of days spent in hospital in the last year of life was greater among high users (persistent: 9 days, non-persistent: 10 days) when compared to non-high users (5 days). High users were also more likely to be admitted to an ICU than non-high users in the last year of life; persistent high users spent a median of 6.9 days in an ICU, while non-persistent high users and non-high users spent 4.1 and 2.7 days, respectively. About 28% of persistent high users had ≥2 ICU admissions, while 8% of non-persistent high users and only 1% of non-high users were admitted more than once. High users were also more likely to be placed in an ALC-designated bed in the last year of life; persistent high users spent a median of 16.0 days in an ALC bed, while non-persistent high users spent 11.0 days and non-high users spent only 7.0 days in ALC. About 11% of persistent high users had ≥2 ALC admissions, while only 2% of non-persistent high users and < 1% of non-high users were admitted more than once.

**Table 2:** Inpatient healthcare use in the last year of life by high user groups

Inpatient Service	Persistent high user (***N*** = 11,375)	Non-persistent high user (***N*** = 78,989)	Non-high user (***N*** = 88,141)	Total (***N*** = 191,310)
# of hospital admissions: mean, median (IQR)	5.0, 4.0 (3–6)	2.8, 2.0 (1–4)	1.8, 2.0 (1–2)	2.5, 2.0 (1–3)
Total # of hospital admissions: N, (Col%)				
1	397 (3.5)	20,569 (26.0)	43,879 (49.8)	66,433 (34.7)
2	1363 (12.0)	20,793 (26.3)	25,891 (29.4)	50,866 (26.6)
3	1941 (17.1)	16,011 (20.3)	11,616 (13.2)	32,288 (16.9)
4	2019 (17.8)	10,084 (12.8)	4468 (5.1)	18,698 (9.8)
≥5	5655 (49.7)	11,532 (14.6)	2287 (2.6)	23,025 (12.0)
Total days in hospital: mean, median (IQR)	20.2,9 (4–23)	19.8,10 (4–23)	7.0,5 (2–9)	15.0,7 (3–16)
**Intensive Care Unit**				
# of admissions to ICU (Col%)				
0	49	77	92	81.1
1	24	15	7	12.3
≥2	28	8	1	6.6
# of admissions to ICU^a^: mean, median (IQR)	2.2, 2.0 (1–3)	1.6, 1.0 (1–2)	1.2, 1.0 (1–1)	1.6, 1.0 (1–2)
Total days in ICU^a^: mean, median (IQR)	13.7, 6.9 (3–15)	7.6, 4.1 (2.0–8.7)	3.7, 2.7 (1.3–4.8)	7.9, 4.1 (1.9–8.6)
**Alternate Level of Care**				
# of admissions to ALC (Col%)				
0	65	86	94	87
1	24	12	5	11
≥2	11	2	< 1	2
# of admissions to ALC^a^: mean, median (IQR)	1.4, 1.0 (1–2)	1.2, 1.0 (1–1)	1.1, 1.0 (1–1)	1.2, 1.0 (1–1)
Total days in ALC^a^: mean, median (IQR)	32.4, 16.0 (7–41)	22.3, 11.0 (5–26)	10.8, 7.0 (3–13)	23.8, 11.0 (5–27)
**Terminal Hospitalization Admission Type**				
Elective (Col%)	12	10	4	7
Length of Stay: mean, median (IQR)	46.7, 28.0 (11–55)	33.3, 20.0 (9–39)	7.2, 5.0 (2–10)	26.5, 13.0 (5–31)
Emergent/Urgent (Col%)	88	90	96	93
Length of Stay: mean, median (IQR)	34.8, 20.0 (8–44)	32.1, 21.0 (9–40)	7.0, 5.0 (2–10)	18.6, 9.0 (3–22)
**In-hospital Interventions:** N, (Col%)				
Mechanical ventilation	2966 (26%)	22,947 (29%)	13,058 (15%)	40,510 (21%)
Cardiopulmonary resuscitation	685 (6%)	4604 (6%)	4235 (5%)	10,040 (5%)
Defibrillation	232 (2%)	2066 (3%)	1406 (2%)	3863 (2%)
Dialysis	1519 (13%)	6886 (9%)	1608 (2%)	10,492 (5%)
Percutaneous coronary intervention	55 (< 1%)	842 (1%)	732 (1%)	1661 (1%)
Feeding tube	590 (5%)	4000 (5%)	869 (1%)	5586 (3%)
Blood transfusion	33 (< 1%)	88 (< 1%)	25 (< 1%)	153 (< 1%)
Bronchoscopy	277 (2%)	2207 (3%)	278 (< 1%)	2794 (1%)
Any intervention^ab^	4120 (36%)	27,962 (35%)	16,428 (19%)	50,681 (26%)

^a^among those who used the service at least once during the last year of life

^b^ received at least one of the following: mechanical ventilation, cardiopulmonary resuscitation, defibrillation, dialysis, percutaneous coronary intervention, feeding tube, blood transfusion, bronchoscopy

Moreover, we found that persistent and non-persistent high users were more likely to receive in-hospital interventions, compared to non-high users, such as mechanical ventilation (26 and 29% vs. 15%, respectively), dialysis (13 and 9% vs. 2%, respectively) and feeding tubes (5 and 5% vs. 1%, respectively). About 36% of persistent and non-persistent high users received at least one intervention, compared with only 19% of non-high users.

### Costs

Overall, the study cohort incurred over $4.7 billion in direct inpatient spending during the last year of life (Table 3). We found that persistent and non-persistent high users in the last year of life, who comprised 47% of the study population, accounted for more than 80% of the overall acute-care costs. About 11% of the $4.7 billion stemmed from persistent high users, who account for only 6% of the study population. In contrast, non-high users, who comprised of 46% of the study population, contributed to 15% of all acute-inpatient costs.

**Table 3:** Acute-care costs in the last year of life by high user groups

	Persistent high user (***N*** = 11,375)	Non-persistent high user (***N*** = 78,989)	Non-high user (***N*** = 88,141)
**% of Population**	6	41	46
**Mean**	$47,384	$42,654	$8134
**Median**	$28,239	$29,010	$6163
**Minimum**	$1002	$699	$516
**Maximum**	$838,166	$12,162,018	$72,783
**10th percentile**	$4057	$4809	$1541
**25th percentile**	$11,536	$13,266	$2956
**75th percentile**	$55,177	$50,064	$11,457
**90th percentile**	$106,904	$86,703	$17,723
**95th percentile**	$158,472	$125,558	$21,576
**99th percentile**	$330,339	$271,798	$29,128
**Total Cost**	$538,990,725	$3,369,173,109	$716,930,080
**% of Total Cost**	11.4	71.2	15

The median cost for persistent and non-persistent high users was 4.5 times greater than that of non-high users. In terms of highest acute-care cost, the top 1% (99th percentile) of persistent and non-persistent high users exhibited cost values that were roughly 10 times greater than their non-high user counterparts.

### Differences in high users across Canada

**Fig. 1:**
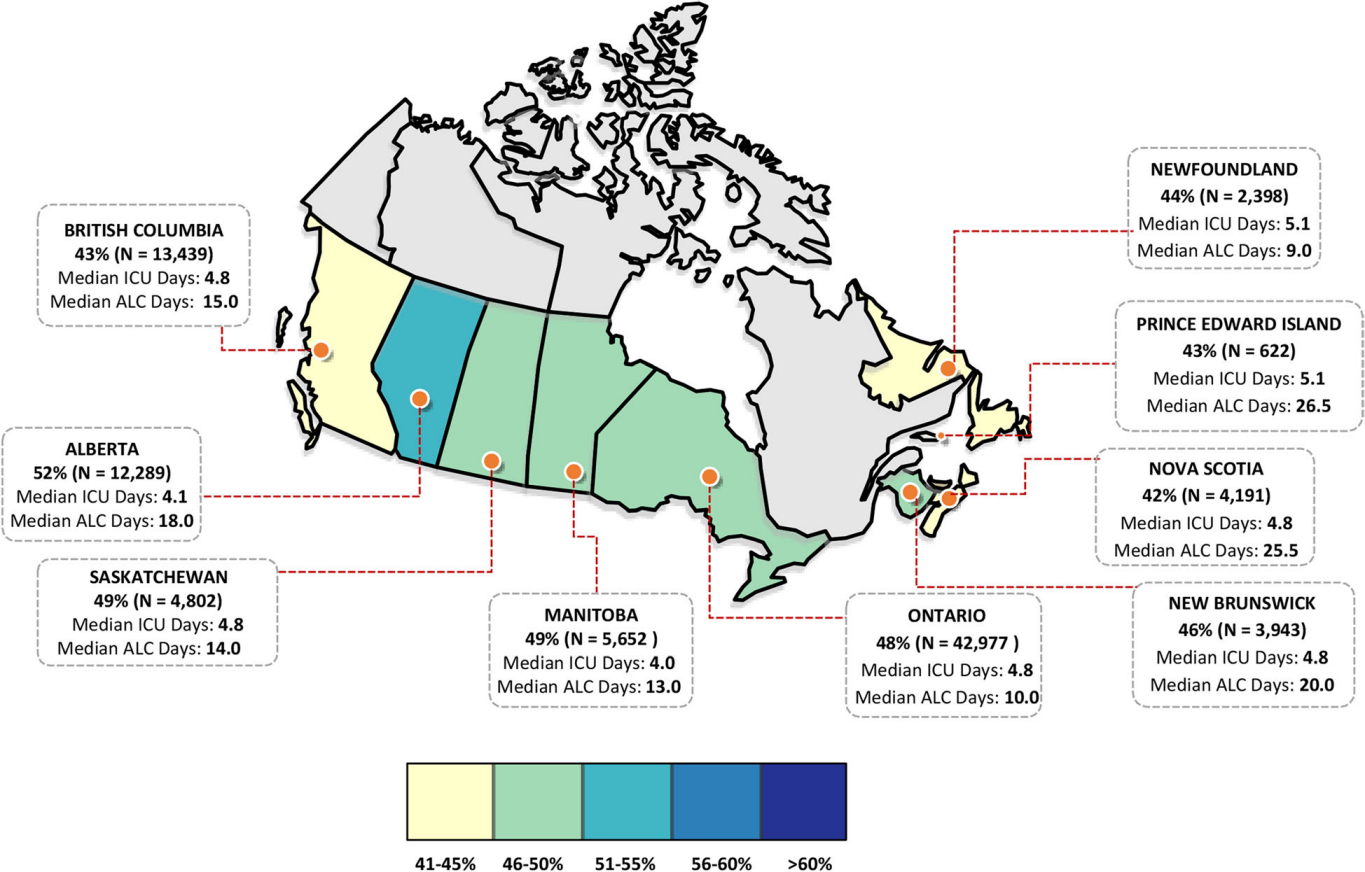
Map displaying the proportion of high users across Canada and their median ICU and ALC days in the last year of life. Map adapted with permission from Templates from yourfreetemplates.com [26]

Figure 1 depicts a colour-coded map comparing decedents flagged as high users (persistent plus non-persistent) in the last year of life across Canada. Among the provinces, Alberta had the highest proportion (52%) of individuals flagged as high users in the last year of life, while Nova Scotia had the least (42%). When we examined the length of stay in ICUs, the median number of days spent in an ICU were highest among high users residing in Newfoundland and Prince Edward Island (5.1 days for both). In contrast, high users in Manitoba and Alberta had the fewest median ICU days in the last year of life (4.0 and 4.1 days, respectively).

Considerable differences were observed in ALC use across Canadian provinces. High users in Prince Edward Island (26.5 days) and Nova Scotia (25.5 days) had the highest median length-of-stay in an ALC bed, while those in Newfoundland (9.0 days), and Ontario (10.0 days) had the lowest median ALC days.

## Discussion

In this pan-Canadian population-based cohort study, we characterize inpatient adult decedents by their pattern of acute-care use, and describe and contrast their healthcare use and acute-care costs in the last year of life. Compared to non-high users, we found that high users were more likely to have multiple hospitalizations, as well as multiple ICU admissions and placements into ALC, with considerably greater lengths of stay. Consumption of these resources were markedly greater for persistent high users than other groups. High users also underwent in-hospital interventions more often than non-high users. Persistent high users, a relatively small group, accounted for a disproportionate amount of inpatient resource use and costs. Despite representing only 47% of the total inpatient study population, persistent and non-persistent high users accounted for more than 80% of overall acute-care costs.

To date, few studies have examined the characteristics and inpatient healthcare use of in-hospital decedents across high-cost user groups presented in this paper, specifically at the end of life. Our findings are similar to that of a population-based study of Western Australia examining how high-cost users (defined as the top 5% of users) of inpatient care differ from other users in the last year of life which found that: hospital resource use was disproportionately concentrated among high users who also accounted for almost 40% of inpatient costs, high users had higher comorbidity scores, and many were hospitalized for chronic conditions such as end-stage renal disease, cancer, angina, and congestive heart failure [27]. Other previous research assessing patterns of healthcare use and expenditures among high-cost inpatient survivors and decedents in Ottawa, Ontario, also found that persistently high-cost patients were more likely to have multiple ICU admissions and ALC placements, and these individuals exhibited significantly greater inpatient spending compared to other groups [18]. Moreover, consistent with several studies of high-cost users identified in a review by Wammes et al. [19], our results confirm the high burden of comorbidities, the high utilization of inpatient resources, and significant impact of inpatient care costs among high users [15, 19, 28, 29].

Similar to previous study findings from Medicare populations [30], we found that the prevalence of congestive heart failure, chronic obstructive pulmonary disease, renal failure and complex cases of diabetes were high among persistent high users. These highly prevalent conditions often lead to intensive outpatient management, which may include care from more than one specialty [30, 31]. This is especially true for those suffering from end-stage renal failure, as these patients usually require several services, such as dialysis, and specialist visits. Once patients with these kinds of conditions become a high-cost user, it is often challenging to reduce their expenditures, as spending for these patients typically increases in following years [30]. These findings of highly prevalent conditions, coupled with the high proportion of elderly individuals and high rate of comorbidities observed, demonstrate that persistent high users are often more medically complex than non-persistent high users and non-high users. Moreover, persistent high users have been found to be older than non-persistent high users in some previous studies [32–34], but younger in others [18, 30, 35]. Interestingly, in our study, we found that high users generally comprised those of older age, but a greater proportion of persistent high users were in the younger age categories when compared to non-persistent high users and non-high users (32% vs. 22% vs. 16%, respectively, were under 65).

High users identified in our study also experienced frequent hospitalizations and ICU admissions near the end of life. Notably, more than four-fifths of persistent high users, and almost half of non-persistent high users experienced ≥3 hospital admissions. Moreover, almost one-third of persistent high users experienced multiple ICU admissions. Use of these resources may explain the disproportionately high amount of healthcare expenditures among the high user groups. Further, many of these patients are likely at high risk of poor short- and long-term health outcomes as well [36]. These adverse events may potentially be mitigated by introducing high quality, early palliative and community-based care, which – in the context of overly aggressive treatments – has shown positive results including reduced inpatient and ICU visits, reduced length-of-stay and direct costs, and improved quality of communication [37–40].

We also found that approximately 11% of persistent high users had multiple ALC admissions in the last year of life. This finding may hint at inefficiencies within our healthcare system – as ALC service use is often considered a marker of inefficient use of hospital resources [25, 41] – to effectively move high-risk patients out of costly and resource-intensive acute-inpatient settings, but can also be seen as an opportunity to improve the availability of end-of-life community-based supports that would allow patients to be safely and effectively discharged from hospital. Further research should focus on identifying the major barriers hindering efficient transitions out of acute-care and should investigate the various predisposing, enabling, and need factors that are associated with high-cost and intensive use of inpatient services. Notably, we also found considerable differences in ALC use across Canadian provinces, with the median number of ALC days ranging from 9.0 (Newfoundland) to 26.5 (Prince Edward Island) in the last year of life. These variations may be a function of differences in patient needs, system capacity, and availability of post-acute-care resources such as long-term care, home care supports, and other initiatives to support patients and their informal caregivers in the community. However, results showing provincial differences should be interpreted with caution as it remains unclear to what extent these variations reflect real differences in patient care, availability of community resources or inconsistencies in data collection/documentation practices of ALC across the country. Future research could examine data comparing access to home and community care services among high users and non-high users to better understand the relationship between the supply of post-acute-care resources and the use of inpatient services, such as ALC.

### Limitations

Our study also has several limitations. Firstly, we did not capture healthcare use and costs outside of the hospital setting. However, we do provide information from acute-care settings, which are the most often reported primary expenditure category for high-cost patients at the end of life. Second, the cross-sectional nature of this study limit conclusions that can be drawn for causality. Further, our analysis was limited to in-hospital decedents. Also, our case definitions might not capture some of the nuances of the patient experience; patients hospitalized for a particular condition may be deemed ‘non-high users’ simply because they die shortly after admission, while patients deemed as ‘high users’ may have achieved this status as a result of having survived long enough to incur considerable costs, and thus meeting the criteria to be flagged as such.

## Conclusion

In conclusion, we found that high users often present with medically complex conditions, high needs, and use a disproportionate amount of inpatient healthcare resources at the end of life. A greater understanding of the characteristics and circumstances that lead to persistently (or non-persistently) high use of inpatient services may help improve strategies that could prevent hospitalizations and off-set current healthcare costs while improving patient outcomes.

## Supplementary Information

**Additional file 1: Supplemental Table 1.** Cohort selection. **Supplemental Table 2.** Dynamic cohort methodology. **Supplemental Table 3**: Cohort characteristics in the last year of life among patients flagged as ‘high-cost in the year prior to death, but not in the death year’. **Supplemental Table 4.** Inpatient healthcare use in the last year of life among patients flagged as ‘high-cost in the year prior to death, but not in the death year’. **Supplemental Table 5.** Acute care costs in the last year of life among patients flagged as ‘high-cost in the year prior to death, but not in the death year’.

## Data Availability

While data sharing agreements prohibit the research team and CIHI from making the dataset publicly available, access may be granted to those who meet pre-specified criteria for confidential access. The full dataset creation plan and underlying analytic code are available from the authors upon request.

## Abbreviations

ICU: Intensive Care Unit
ALC: Alternate Level of Care
CIHI: Canadian Institute for Health Information
DAD: Discharge Abstract Database
