# Supplementary material for: Describing the characteristics and healthcare use of high-cost acute care users at the end of life: a pan-Canadian population-based study
Source: BMC Health Serv Res. 2020 Oct 31;20:997. doi: 10.1186/s12913-020-05837-8 (PMC7603700; doi:10.1186/s12913-020-05837-8)
Supplement: Supplementary file 1 — Additional file 1: Supplemental Table 1. Cohort selection. Supplemental Table 2. Dynamic cohort methodology. Supplemental Table 3: Cohort characteristics in the last year of life among patients flagged as ‘high-cost in the year prior to death, but not in the death year’. Supplemental Table 4. Inpatient healthcare use in the last year of life among patients flagged as ‘high-cost in the year prior to death, but not in the death year’. Supplemental Table 5. Acute care costs in the last year of life among patients flagged as ‘high-cost in the year prior to death, but not in the death year’. [file 12913_2020_5837_MOESM1_ESM.docx]

Supplemental Table 1: Cohort selection

| **Step** | **N** | **Description** |
| --- | --- | --- |
| 1 | 336,471 | All CIHI-DAD hospital death records available between fiscal years 2011/12 and 2014/15 |
| 2 | 336,429 | Exclude those with duplicate records or multiple death dates |
| 3 | 244,630 | All cases after CIHI-DAD record linkage to valid (non-missing RIW) CIHI-Dynamic Cohort records between fiscal years 2011/12 and 2014/15 |
| 4 | 191,791 | Exclude those that did not have at least two consecutive years of available data |
| 5 | 191,489 | Exclude those with missing and/or invalid health card data |
| 6 | 191,465 | Exclude those with missing age and/or sex |
| 7 | 191,455 | Exclude those that were not a Canadian resident |
| 8 | 191,310 | Exclude those with an acute-care length of stay ≥ 360 days |

Supplemental Table 2: Dynamic cohort methodology

| **Dynamic Cohort Methodology** | In 2019, the Canadian Institutes of Health Research partnered with CIHI to create a dynamic cohort of complex, high system users. The Dynamic Cohort is an inpatient dataset that contains several subset cohorts encompassing various definitions of “complex, high system users”. In this analysis, we used a cohort of complex, high system users defined based on the highest acute care costs; these high users were defined as the top 10% of highest cumulative acute care cost patients in each fiscal year.  The dynamic cohort was created using CIHI’s Cost of a Standard Hospital Stay (CSHS) indicator for acute inpatient hospitalization data from CIHI’s DAD. The CSHS indicator measures the relative cost-efficiency of a hospital’s ability to provide acute inpatient care. The indicator compares a hospital’s overall acute inpatient care expenditures to the number of acute inpatient weighted cases. The result is the hospital’s average full cost of treating the average acute inpatient. The CSHS values were multiplied by the Resource Intensity Weight (RIW) costing data available in the DAD, providing an estimate of the cost of an acute inpatient hospital stay for each individual patient. The cost estimates are adjusted between provinces to account for differences in data structure and quality.  More information: <https://cihr-irsc.gc.ca/e/50129.html> |
| --- | --- |

Supplemental Table 3: Cohort characteristics in the last year of life among patients flagged as ‘high-cost in the year prior to death, but not in the death year’

|  | **(N=12,805)** | |
| --- | --- | --- |
| **Characteristics** | **N** | **Col%** |
| **Sex** | | |
| Female | 6051 | 47 |
| Male | 6754 | 53 |
| **Age** | | |
| 18-44 | 342 | 3 |
| 45-54 | 679 | 5 |
| 55-64 | 1578 | 12 |
| 65-74 | 2631 | 21 |
| 75-84 | 3946 | 31 |
| ≥85 | 3629 | 28 |
| **Rurality** | | |
| Urban | 11233 | 88 |
| Rural | 1572 | 12 |
| **Elixhauser Comorbid Conditions** | | |
| Cardiac Arrhythmia | 2251 | 18 |
| Congestive Heart Failure | 2971 | 23 |
| Chronic Obstructive Pulmonary Disease | 2357 | 18 |
| Depression | 198 | 2 |
| Diabetes - Complicated | 3074 | 24 |
| Diabetes - Uncomplicated | 854 | 7 |
| Hypertension - Complicated | 112 | 1 |
| Hypertension - Uncomplicated | 2922 | 23 |
| Hypothyroidism | 240 | 2 |
| Liver Disease | 659 | 5 |
| Lymphoma | 426 | 3 |
| Metastatic Cancer | 2439 | 19 |
| Other Neurological Disorders | 795 | 6 |
| Psychoses | 50 | 0 |
| Renal Failure | 1344 | 10 |
| Tumor (Solid Tumor without Metastasis) | 3184 | 25 |
| **# of Comorbidities** | | |
| 0 | 1218 | 10 |
| 1-2 | 6671 | 52 |
| 3-5 | 4608 | 36 |
| 6+ | 308 | 2 |

Supplemental Table 4: Inpatient healthcare use in the last year of life among patients flagged as ‘high-cost in the year prior to death, but not in the death year’

|  | **(N=12,805)** |
| --- | --- |
| # of hospital admissions: mean, median (IQR) | 3.6, 3.0(2-5) |
| Total # of hospital admissions: N, (Col%) |  |
| 1 | 1588(12.4) |
| 2 | 2819(22.0) |
| 3 | 2720(21.2) |
| 4 | 2127(16.6) |
| ≥5 | 3551(27.7) |
| Total days in hospital: mean, median (IQR) | 13.2,7(3-14) |
| **Intensive Care Unit** |  |
| # of admissions to ICU (Col%) |  |
| 0 | 61 |
| 1 | 23 |
| ≥2 | 16 |
| # of admissions to ICU‡ :mean, median (IQR) | 1.7, 1.0(1-2) |
| Total days in ICU‡: mean, median (IQR) | 8.3, 5.0(2.3-9.9) |
| **Alternate Level of Care** |  |
| # of admissions to ALC (Col%) |  |
| 0 | 65 |
| 1 | 28 |
| ≥2 | 7 |
| # of admissions to ALC‡: mean, median (IQR) | 1.2, 1.0(1-1) |
| Total days in ALC‡: mean, median (IQR) | 35.2, 19.0(7-43) |
| **Terminal Hospitalization Admission Type** |  |
| Elective (Col%) | 5 |
| Length of Stay: mean, median (IQR) | 9.0, 6.0(3-13) |
| Emergent/Urgent (Col%) | 95 |
| Length of Stay: mean, median (IQR) | 7.2, 5.0(2-10) |
| **Interventions:** N, (Col%) |  |
| Mechanical ventilation | 1539 (12%) |
| CPR | 516 (4%) |
| Defibrillation | 159 (1%) |
| Dialysis | 479 (4%) |
| PCI | 32 (0%) |
| Feeding tube | 127 (1%) |
| Blood transfusion | 7 (0%) |
| Bronchoscopy | 32 (0%) |
| Active Intervention | 2171 (17%) |

Supplemental Table 5: Acute care costs in the last year of life among patients flagged as ‘high-cost in the year prior to death, but not in the death year’

|  | **(N=12,805)** |
| --- | --- |
| **% of Population** | 7 |
| **Mean** | $8,273 |
| **Median** | $6,236 |
| **Minimum** | $850 |
| **Maximum** | $76,714 |
| **1st percentile** | $1,062 |
| **5th percentile** | $1,233 |
| **10th percentile** | $1,519 |
| **25th percentile** | $2,918 |
| **75th percentile** | $11,673 |
| **90th percentile** | $18,134 |
| **95th percentile** | $22,027 |
| **99th percentile** | $29,990 |
| **Total Cost** | $105,934,485 |
| **% of Total Cost** | 2 |
